# Supplementary figures and images for: Identification and functional analysis of bacteria in sclerotia of Cordyceps militaris
Source: PeerJ. 2021 Nov 25;9:e12511. doi: 10.7717/peerj.12511 (PMC8627653; doi:10.7717/peerj.12511)

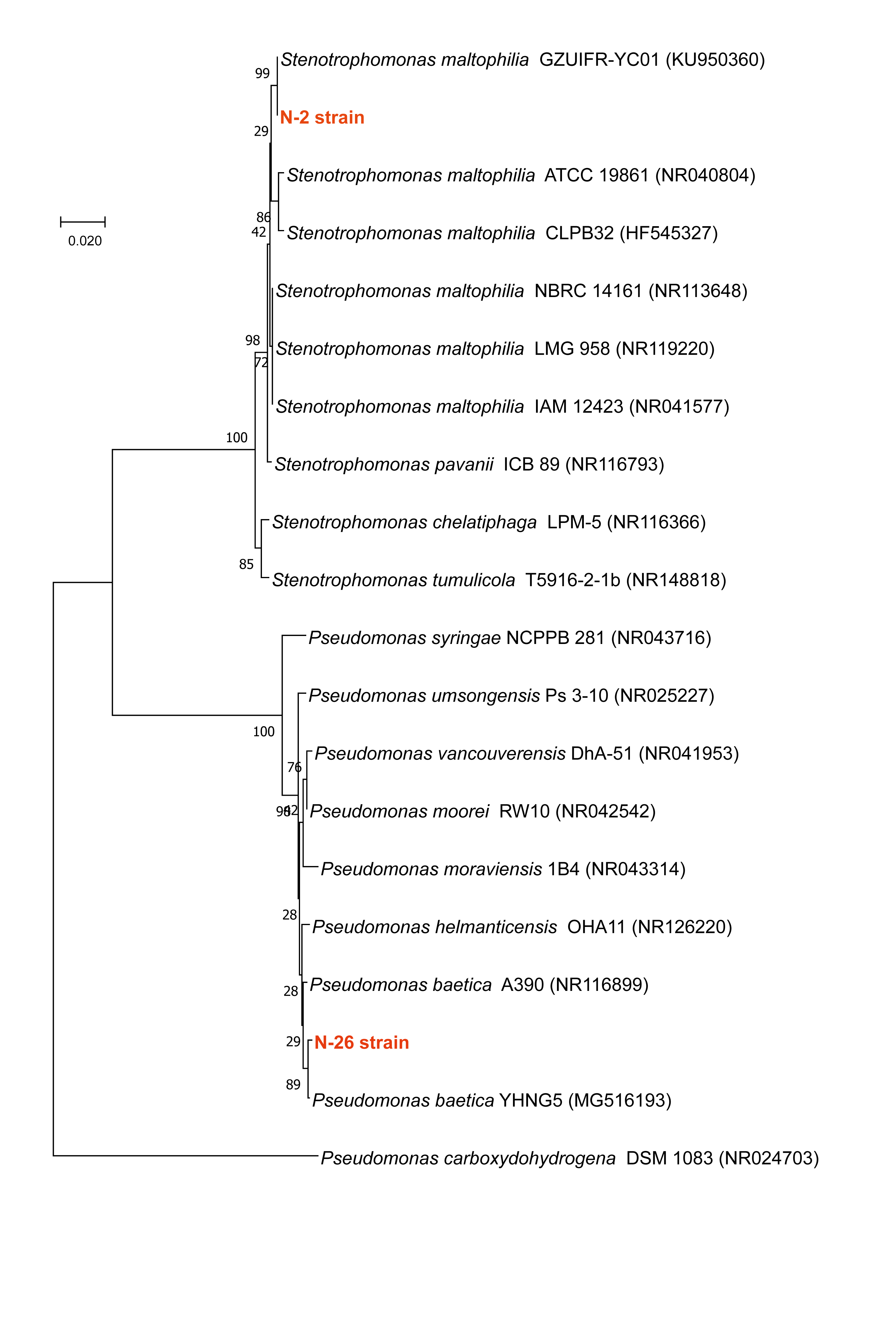

Supplement: Supplemental Information 1 — Note: The number at each branch point is the bootstrap percentage (1000 resamplings). Numbers in parentheses are GenBank accession codes. Bar: 2% sequence divergence. [file peerj-09-12511-s001.png]

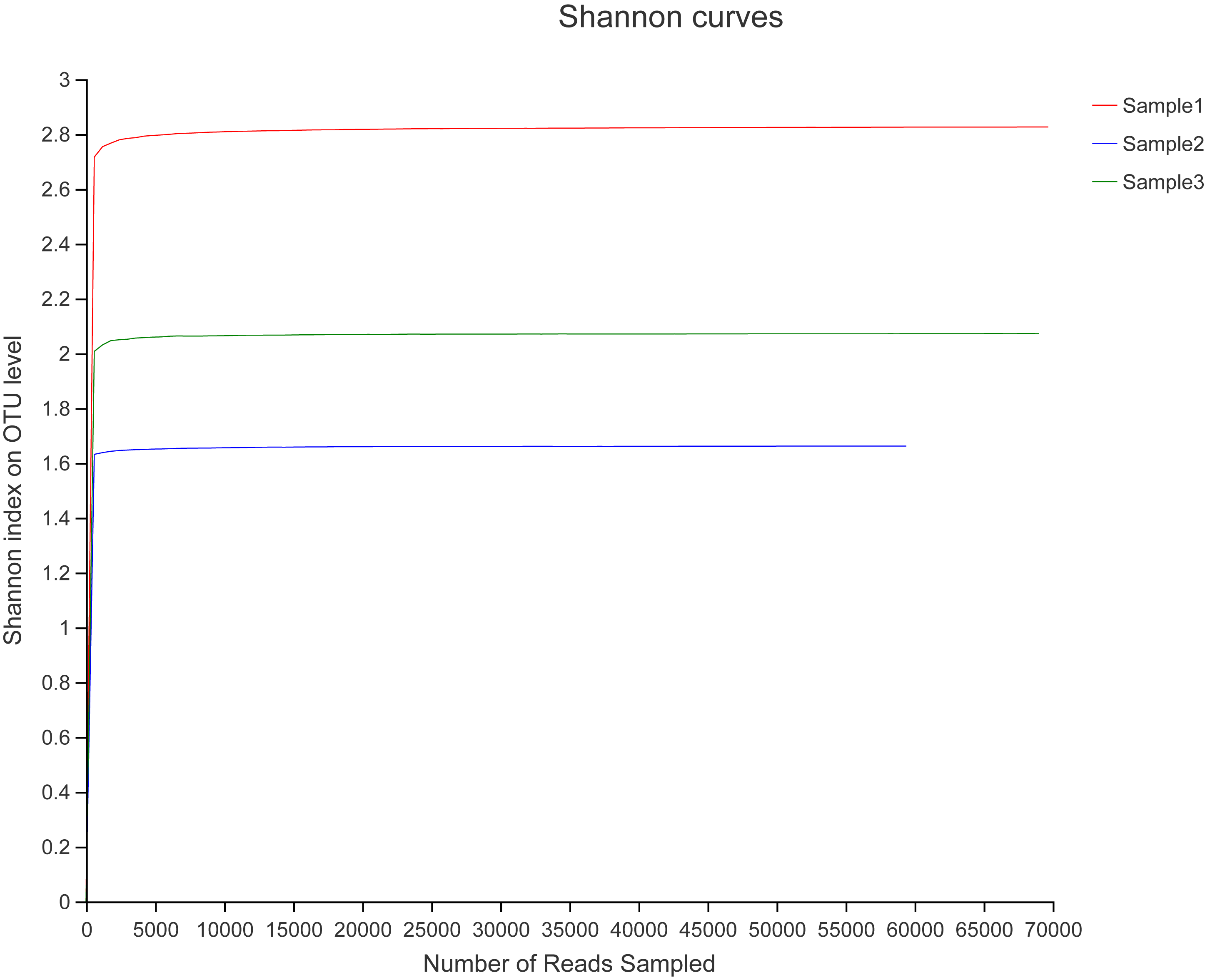

Supplement: Supplemental Information 2 [file peerj-09-12511-s002.png]

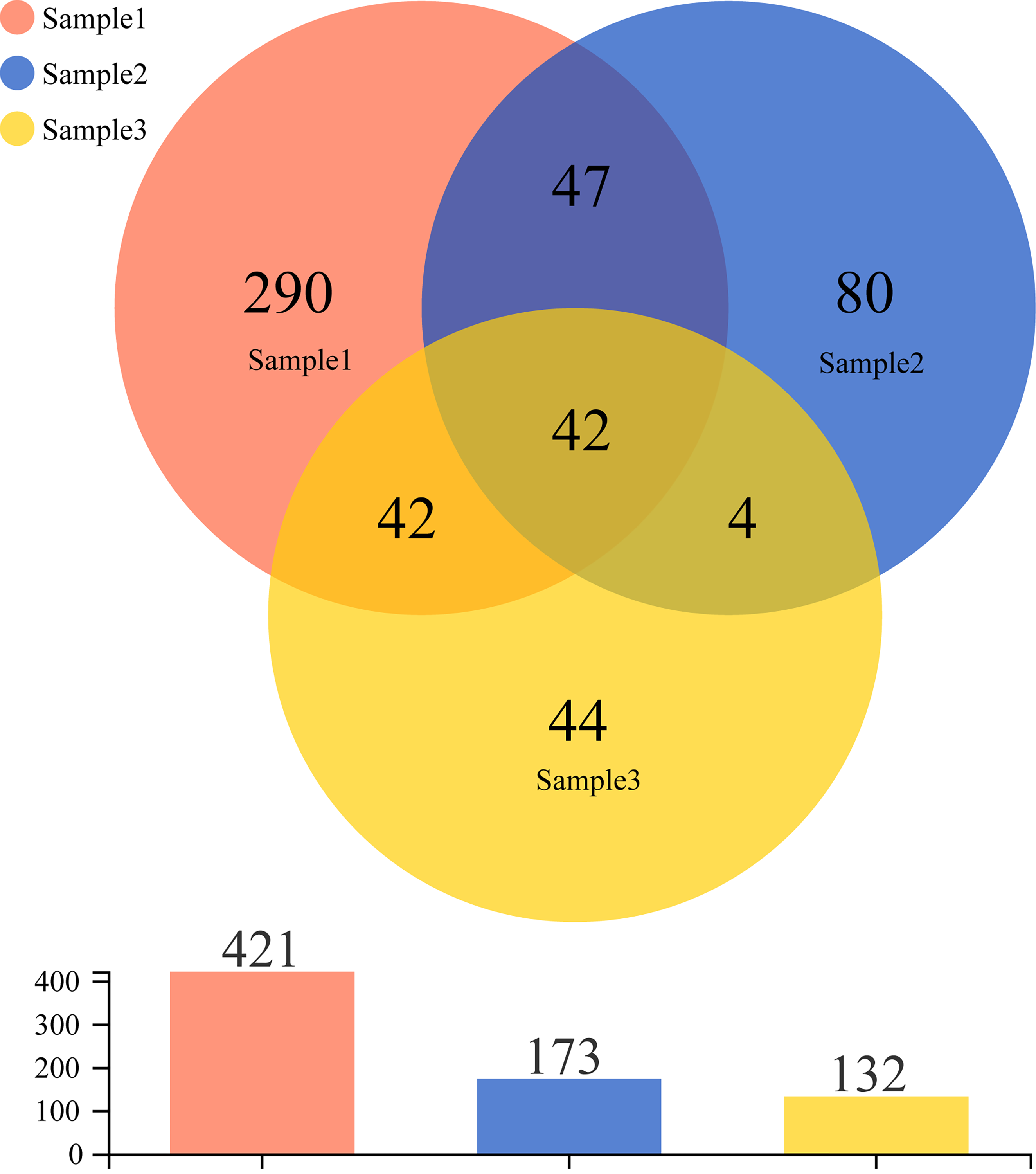

Supplement: Supplemental Information 3 [file peerj-09-12511-s003.png]
