# Supplementary material for: Identification and functional analysis of bacteria in sclerotia of Cordyceps militaris
Source: PeerJ. 2021 Nov 25;9:e12511. doi: 10.7717/peerj.12511 (PMC8627653; doi:10.7717/peerj.12511)
Supplement: Supplemental Information 5 [file peerj-09-12511-s005.docx]

**Supplemental Table S1**. Physiological and biochemical characteristics of strains N-2 and N-26.

| **Indicators** | **Strain** | | **Indicators** | **Strain** | |
| --- | --- | --- | --- | --- | --- |
|  | **N-2** | **N-26** |  | **N-2** | **N-26** |
| Semi-solid Agar | + | + | Mannitol | - | - |
| Gelatin | - | - | Inositol | - | - |
| Ornithine decarboxylase | + | + | Sorbitol | - | - |
| Lysine decarboxylase | + | + | Melibiose | - | - |
| Amino acid decarboxylase | + | + | Adonitol | - | - |
| Simmons citrate | - | - | Raffinose | - | - |
| Hydrogen sulfide | - | - | Xylose | - | - |
| Urease | - | - | Maltose | - | - |
| Peptone hydrolysis | - | - | Lipase | - | - |
| MR test | - | - | D-mannitol | - | - |
| VP test | + | + | Salicin | - | - |
| Phenylalanine | - | - | Aescinoside hydrolyzed | + | - |

Note: “+” means positive, “−” means negative
