# Supplementary material for: Identification and functional analysis of bacteria in sclerotia of Cordyceps militaris
Source: PeerJ. 2021 Nov 25;9:e12511. doi: 10.7717/peerj.12511 (PMC8627653; doi:10.7717/peerj.12511)
Supplement: Supplemental Information 6 [file peerj-09-12511-s006.docx]

**Supplemental Table S2**. Classification, functional prediction and abundance of OTUs in sclerotia of *C. militaris*.

| **OTU ID** | **Taxonomy** | **Function of prediction** | **Abundance** | |
| --- | --- | --- | --- | --- |
| OTU1940 | Allorhizobium-Neorhizobium-Pararhizobium-Rhizobium | None | | 29 |
| OTU1261 | Devosia | None | | 47 |
| OTU1448 | Pseudomonas | aerobic chemoheterotrophy；chemoheterotrophy | | 537 |
| OTU2324 | Comamonas | None | | 713 |
| OTU1490 | Stenotrophomonas | human pathogens；animal parasites or symbionts；nitrate respiration；nitrate reduction；nitrogen respiration；aerobic chemoheterotrophy；chemoheterotrophy； | | 3593 |
| OTU1229 | norank_f__Ilumatobacteraceae | None | | 6 |
| OTU2330 | Pseudomonas | aerobic chemoheterotrophy；chemoheterotrophy | | 32281 |
| OTU1071 | Pedomicrobium | aerobic chemoheterotrophy；chemoheterotrophy | | 33 |
| OTU1534 | Pedobacter | aerobic chemoheterotrophy；chemoheterotrophy | | 20951 |
| OTU1539 | Rhodococcus | ligninolysis | | 11404 |
| OTU2342 | Sphingobacterium | ureolysis | | 11404 |
| OTU2028 | norank_c__KD4-96 | None | | 18 |
| OTU1507 | unclassified_f__Micromonosporaceae | None | | 15 |
| OTU2350 | Sphingopyxis | aerobic chemoheterotrophy；chemoheterotrophy | | 109 |
| OTU1732 | Gaiella | None | | 11 |
| OTU2338 | Bosea | None | | 199 |
| OTU1633 | Ochrobactrum | None | | 2009 |
| OTU1632 | Variovorax | None | | 6207 |
| OTU1243 | Labrys | aerobic chemoheterotrophy；chemoheterotrophy | | 2121 |
| OTU1854 | norank_f__Methyloligellaceae | None | | 22 |
| OTU1479 | Sphingobacterium | None | | 3105 |
| OTU4 | Ralstonia | None | | 11 |
| OTU1179 | Microlunatus | aerobic chemoheterotrophy；chemoheterotrophy | | 24 |
| OTU1047 | Ensifer | nitrate reduction；aerobic chemoheterotrophy；chemoheterotrophy； | | 96 |
| OTU1477 | Luteibacter | None | | 7692 |
| OTU1313 | Phyllobacterium | None | | 3343 |
| OTU1629 | Achromobacter | None | | 12357 |
| OTU2313 | Delftia | None | | 864 |
| OTU1579 | Serratia | fermentation | | 1556 |
| OTU1423 | Rhodococcus | aromatic compound degradation；aliphatic non methane hydrocarbon degradation；hydrocarbon degradation；chemoheterotrophy； | | 16 |
| OTU6 | unclassified_f__Enterobacteriaceae | None | | 26773 |
| OTU2234 | Candidatus_Xiphinematobacter | animal parasites or symbionts | | 7 |
| OTU1387 | Mycobacterium | aerobic chemoheterotrophy；chemoheterotrophy | | 61 |
| OTU2314 | Pseudomonas | aerobic chemoheterotrophy；chemoheterotrophy | | 75 |
| OTU2356 | Rhodopseudomonas | None | | 124 |
| OTU1845 | Allorhizobium-Neorhizobium-Pararhizobium-Rhizobium | nitrogen fixation | | 385 |
| OTU1334 | norank_f__norank_o__IMCC26256 | None | | 15 |
| OTU1529 | Arthrobacter | None | | 24 |
| OTU212 | Ellin6055 | aerobic chemoheterotrophy；chemoheterotrophy | | 20 |
| OTU1060 | Mesorhizobium | None | | 89 |
| OTU2348 | Curtobacterium | aerobic chemoheterotrophy；chemoheterotrophy | | 2312 |
